# Qualitative insights into the experiences of living with moderate-to-severe lower urinary tract symptoms among community-dwelling ageing males

**DOI:** 10.1371/journal.pone.0187085

**Published:** 2017-10-30

**Authors:** Lorna Kwai Ping Suen, Hui Lin Cheng, Simon Kai Wang Yeung, Cypher Ho Au-Yeung, Jillianne Chi Yen Lee, Kathy Kit Ying Ho, Natalie Ming Yan Lau, Cristina Ka Fu Ng, Iris Wai Sze Chan

**Affiliations:** 1 School of Nursing, The Hong Kong Polytechnic Univeristy, HungHom, Hong Kong; 2 Ambulatory Surgery Centre, Tseung Kwan O Hospital, Hong Kong; 3 Department of Surgery, Gleneagles Hong Kong Hospital, Hong Kong; 4 Nursing Department, Hong Kong Sanatorium Hospital, Hong Kong; 5 Operation Theatre, Princess Margaret Hospital, Hong Kong; 6 Department of Medical & Geriatric, Princess Margaret Hospital, Hong Kong; Human Sciences Research Council, SOUTH AFRICA

## Abstract

**Background:**

Lower urinary tract symptoms (LUTS) comprise a highly prevalent chronic condition among the aging male population. Existing literature on the experiences of men with LUTS is scarce given that only a few studies explored medical care-seeking behaviors and coping strategies. The current understanding of the experiences of elderly males with LUTS is considerably limited. Therefore, the present study aimed to identify the experiences of living with moderate-to-severe LUTS among community-dwelling Chinese ageing males and their coping strategies to facilitate the management of LUTS by healthcare providers.

**Methods and findings:**

A qualitative exploratory design using thematic analysis was used. Semi-structured interviews with 24 Chinese ageing males with moderate-to-severe LUTS were conducted. According to the participants, LUTS adversely affect the physical aspects of their daily lives. Most of them were unwilling to seek social support and were even embarrassed to share this topic with their peers. A range of psychological responses could be observed from the participants that range from regarding the condition as a natural life course to loss of one’s self-esteem. Most of the interviewees lacked knowledge and held misconceptions toward LUTS, which prevented them from pursuing medical advice. Most of the participants also sought alternative treatments and developed self-help methods to cope with their symptoms.

**Conclusion:**

LUTS affects the physical and social aspects of sufferers. The findings of this qualitative study can raise awareness about the life experiences, perceptions, misconceptions, and help-seeking behaviors of Chinese elderly with LUTS. Proper health education and advice can be provided for this population.

## Introduction

Lower urinary tract symptoms (LUTS) comprise a complex symptom cluster that encompasses storage (e.g. nocturia), voiding (e.g. weak stream) and post-micturition (e.g. incomplete emptying) symptoms [1]. LUTS is a highly prevalent chronic condition that affects almost half of the world’s population [2]. LUTS prevalence increases with age regardless of gender, but severe LUTS is common among the male population [3]. The effect of LUTS among affected males is significant and includes low quality of life (QoL), high risk of depression, sexual dysfunction and falls [4–7].

Disease development varies across ethnicities. This observation is also true for the LUTS prevalence, which highlights ethnical differences [8]. In the United Kingdom, a study on men aged over 40 years showed that 36.5% of the South Asian group experienced at least one LUTS. By contrast, only 29.0% of the Western group is affected by this problem [9].

Existing literature on the experiences of men with LUTS is scarce because only a few studies explored medical care-seeking behaviour and coping strategies [10–14]. A considerable number of studies focused on the experiences of ageing males with LUTS in the United Kingdom [13,15], the United States [16,17], South Australia [18], New Zealand [19] and Central Scotland [20]. However, information about this health problem is insufficient in Asian countries [21]. Given that underlying ethnic, social and educational backgrounds may affect the attitudes and coping strategies of patients [10–12], this study aimed to identify the experiences of living with moderate-to-severe LUTS among community-dwelling Chinese ageing males and their coping strategies to facilitate LUTS management by healthcare providers.

## Methods

### Design

A qualitative exploratory descriptive design using thematic analysis was adopted in this study; the analysis was guided by the philosophy of naturalistic enquiry [22]. Through this approach, researchers were expected to uncover various aspects of the studied phenomenon and underlying process, particularly when less is known about this phenomenon [23]. For the purpose of this study, this design allowed us to explore and describe how ageing Chinese males live and cope with moderate-to-severe LUTS in their daily lives. This study ultimately aimed to generate new knowledge in the field. Individual face-to-face interviews were conducted because of the sensitive nature of LUTS, which can relate to sexuality.

### Setting and participants

Participants were recruited from four elderly community centres in Hong Kong that offer social support for community-dwelling elders [24]. These participants were recruited from this setting because it can provide researchers’ access to community-dwelling elders. Purposive sampling was employed to recruit appropriate participants who would be able to provide rich information about their experiences with LUTS and obtain a mixture of participants with moderate or severe LUTS symptoms. LUTS severity in each participant was assessed using the Chinese version of the International Prostate Symptom Score (IPSS), a questionnaire for evaluating symptom severity [25]. The participants completed the IPSS questionnaire after the researcher explained the items. The IPSS consists of seven Likert-scale questions (with scores ranging from 0 ‘not at all’ to 5 ‘almost always’) related to LUTS. The total score ranged from 0 to 35 (1–7 = mildly symptomatic, 8–19 = moderately symptomatic and 20–35 = severely symptomatic). Three questions involved filling problems (daytime frequency and nocturnal urgency), whereas four questions assessed voiding problems (emptying, intermittency, weak stream and straining). The instrument also included one question that evaluates the effect of urinary symptoms on QoL. The scores ranged from 0 to 6 (0 = delighted, 1 = pleased, 2 = mostly satisfied, 3 = mixed, about equally satisfied and dissatisfied, 4 = mostly dissatisfied, 5 = unhappy and 6 = terrible). The participants were asked to urinate into a specific funnel connected to a portable uroflowmeter (FloPoint Elite Uroflow System). This instrument was used to measure the maximum urinary flow rate (Qmax) per second and evaluate the severity of urinary obstruction. Only the participants with Qmax<15 mL/s were recruited.

Participants were considered eligible if they were Chinese men aged over 60 years old with moderate-to-severe LUTS (IPSS score≥12) and whose maximum urinary flow rate was Qmax<15 mL/s for at least six months [25]. Participants with urinary tract infection or a history of bladder surgery, prostate cancer or psychiatric illness were excluded.

### Data collection

The participants were screened and recruited by the trained researcher (SKWY). After consenting to join the study, each participant was invited to an individual face-to-face interview at home or at the elderly centre. Each semi-structured interview was limited to one participant because of the sensitivity of the topic. The average length of each interview session was approximately 1 h.

A semi-structured interview guide was used to aid the interview and stimulate discussion around the topic of interest. The guide was developed based on the literature review and refined after discussions with the research team (Table 1). Open-ended and probing questions were used to enable the participants to expand their responses and explanations, thereby allowing the researchers to obtain significant data [23]. Probing questions are usually follow-up questions when additional information is needed. Two researchers were involved in each interview, and field notes were collected. Demographic data, including age, occupation, marital status and length of time living with the symptoms, were obtained. Interviews were audio-recorded after obtaining the consent of participants. Data saturation was reached at the 24th participant when no new information was obtained [26].

**Table 1:** Interview guide.

1. Can you describe the urinary symptoms that you are experiencing?
2. How severe are your urinary symptoms?
3. What are the impacts of LUTS in your life? (physically, daily life, psychologically)
4. How much do you understand about LUTS?
5. What do you think about the causes of the symptoms?
6. Where did you get the information on LUTS?
7. Do you have any worry about the progression of LUTS?
8. Do you think it is necessary to seek medical consultation for the problems? Why or why not?
9. Have you performed anything to alleviate the symptoms?

### Data analysis

Interviews were conducted in Cantonese and audio-recorded and transcribed verbatim for subsequent data analysis. Data were coded using thematic analysis with Braun and Clarke’s approach [27]. This method is systematic and rigorous in identifying, analysing and reporting patterns from qualitative data chosen in relation to the research aim and objectives [27]. For the purpose of this study, we analysed data based on the following study objective: to identify the experiences of living with moderate-to-severe LUTS among community-dwelling ageing Chinese males and their coping strategies to facilitate the management of LUTS by healthcare providers. Braun and Clarke’s analytical approach involves six steps, namely, a) data familiarisation by repeated reading, b) generating initial codes, c) searching for themes, d) reviewing potential themes, e) defining and naming themes and f) producing the report. Three research team members (HLC, SKWY and CHAY) worked together to develop a coding scheme by comparing and contrasting the findings across the interview data. Disagreements or discrepancies in the coding scheme were resolved through group discussions until a consensus was reached. Selected quotes were translated to English so that they can be cited in the publication.

### Trustworthiness

The trustworthiness, including credibility, transferability, dependability and confirmability of this study, was established by adhering to evaluative criteria by Lincoln and Guba [28]. Credibility refers to confidence in the truth of the data. For this study, we established credibility by the following techniques including prolonged engagement with participants and triangulation across different researchers. Through prolonged engagement, participants built trust with the researchers, and they felt comfortable to share their experiences during the interview. For triangulation across different researchers, the data were analysed using more than two researchers to allow multiple interpretations and expand the depth of insights into the data, consequently enhancing the credibility of data analysis. The first author (LKPS) regularly discussed with the study team and randomly selected cases to ensure consistency between data and researchers’ interpretations. Transferability refers to the generalisability of the data. Transferability was achieved by purposively sampling appropriate participants who were able to provide thick and rich descriptions of the study phenomenon. Dependability reflects the consistency of findings. We used a semi-structured interview guide to aid the interview and stimulate discussion around the topic of interest. Audit trails for both study processes and products including audio-recording files, transcripts, data deduction and analysis tables and other discussion notes were kept to allow for verification by the first author (LKPS). Confirmability refers to the neutrality of the findings. During data collection and analysis, the researchers disregarded their knowledge, beliefs, values and experiences about LUTS to avoid researcher bias. Audit trails and reflective journals were applied to enhance the study confirmability.

### Ethical consideration

Ethical approval was obtained from the Human Subjects Ethics Subcommittee of the Hong Kong Polytechnic University [HSEARS20140515003]. Consent was obtained before audio-recording the interviews. The interviews were held in locations where absolute privacy was guaranteed. To ensure the confidentiality of the collected data, the research team restricted data access to the group alone. Participation was on a voluntary basis, and the participants could withdraw from the study at any time.

## Results

Twenty-four participants were interviewed, and data saturation was reached. Fifteen men presented moderate symptoms, and nine men presented severe symptoms. The IPSS scores of the participants ranged from 12 to 35 (mean = 18.71), with a mean Qmax of 9.93 mL/s. The mean age of the participants was 75.21 years (range = 65 to 87 years). The average number of years living with LUTS was 6.38 years (range = 1 to 29), generally with unsatisfactory QoL (mean = 3.17). Majority of the participants (*n =* 15) received education up to the primary school level, and nearly all of them were married (*n* = 23). Table 2 presents the demographic and clinical characteristics of the participants.

**Table 2:** Summary of the demographic characteristics of the participants.

Participant Number	Age	LUTS Duration (years)	Marital status	Education Level	Employment status	IPSS (scores)	QoL	Qmax
P1	69	10	Widowed	Bachelor	Retired	Moderate (17)	4	11
P2	71	12	Married	Primary	Retired	Moderate (19)	2	10.4
P3	87	29	Married	Primary	Retired	Moderate (14)	1	11.4
P4	67	3	Married	Secondary	Retired	Moderate (17)	3	13
P5	81	15	Co-habited	Primary	Retired	Severe (20)	3	5
P6	84	10	Married	Primary	Retired	Severe (26)	3	6
P7	75	1	Married	Primary	Retired	Moderate (12)	3	11
P8	80	3	Widowed	Primary	Retired	Moderate (13)	1	14
P9	74	2.	Married	Secondary	Retired	Moderate (12)	3	8
P10	74	7	Single	Primary	Retired	Moderate (16)	4	13
P11	81	3	Married	Primary	Retired	Moderate (15)	3	6
P12	69	1	Widowed	Primary	Retired	Severe (20)	3	14
P13	83	5	Married	Primary	Retired	Severe (35)	6	5
P14	65	4	Married	Primary	Part-time	Moderate (14)	4	5
P15	71	3	Married	Primary	Retired	Moderate (15)	3	6
P16	67	2	Married	Secondary	Retired	Severe (20)	3	10
P17	79	2	Married	Secondary	Retired	Moderate (12)	2	13
P18	75	5	Married	Secondary	Retired	Severe (29)	4	13
P19	76	3	Married	Primary	Retired	Severe (28)	5	8
P20	82	7	Married	Secondary	Retired	16 (Moderate)	3	13.2
P21	77	3	Married	Secondary	Retired	24 (Severe)	3	9.9
P22	80	3	Married	Secondary	Retired	12 (Moderate)	2	12.3
P23	71	10	Married	Primary	Retired	29 (Severe)	6	8.4
P24	67	10	Married	Primary	Retired	14 (Moderate)	2	11.6

IPSS score: 8–19: moderately symptomatic; 20–35: severely symptomatic. Only moderate-to-severe LUTS (IPSS score > = 12) were recruited; QoL: Quality of life, with the score ranging from 0 to 6(0 = delighted, 1 = pleased, 2 = mostly satisfied, 3 = mixed, about equally satisfied and dissatisfied, 4 = mostly dissatisfied, 5 = unhappy, 6 = terrible); Qmax: maximum urinary flow rate per second. Only participants with Qmax< 15 mL/s were recruited.

The experiences of participants living with LUTS were grouped into three categories as follows: (1) effect of LUTS, (2) perceptions and misconceptions and (3) managing LUTS (S1 Table). The consolidated criteria for reporting qualitative research (COREQ) were used, which consisted of a 32-item checklist for interviews (S2 Table) [29].

### 1. Effect of LUTS

#### 1.1. Frequent uncontrolled urinary habit

Among the different LUTS symptoms, the most bothersome complaints of the participants included frequent urination, urge incontinence, intermittent and slow urination and incomplete emptying. Ten participants experienced frequent urination during day time, which was manifested in urination every less than 2 h.

Most of the time, I feel an urgent need to pee, and I always get my pants wet when I take off my pants too slowly. (P7, moderate LUTS)

If I feel an urgent need to pee, I can’t hold it. I have to pee before getting on the bus. If there was a traffic jam, I would need to hold [urination] for one hour. I am fine with one hour only. (P8, moderate LUTS)

My urinal flow often slows down and eventually stops. Then, I massage my penis and use force to push it [urine] out. Sometimes, there is something inside blocking my urethra. My urinal flow may suddenly stop, and then starts dripping. Once you think it [urination] is done, it starts to drip again. (P6, severe LUTS)

The most troublesome symptom is incomplete emptying. I find it bothersome, especially when I am on the street and in public areas. Sometimes, urine suddenly flows out, causing my pants to get wet. (P9, severe LUTS)

Nocturia was a common symptom cited by all participants. They explained having to get up to urinate at least three to four times a night. Three participants with severe nocturia reported needing to urinate six to seven times a night. Sleep quality was affected in each case.

I need to wake up five to six times a night to urinate. Last night, I still had to urinate even after urinating six to seven times. Usually, I must wake up every 1–1.5 hours (P20, moderate LUTS)

#### 1.2. Avoidance of social activities and a strain on relationship

Some of the participants felt that LUTS affected their social activities and led to avoidance of social situations, long-distance travel and other events that lack easily accessible toilets.

I want to go somewhere far, but I dare not to. Finding a toilet (in a public area) is very troublesome. Sometimes, the elderly center has activities, such as travelling, but I dare not join any of these activities. (P21, severe LUTS)

The symptoms also affected the peer relationships of some of the participants. Most of them coped with the problems on their own and were unwilling to seek social support because they viewed LUTS as an embarrassing topic to share with others.

No, we never talk about this problem. We usually do not talk about personal issues. It is embarrassing. (P20, moderate LUTS)

The unwillingness to share with others, including one’s family members, was commonly observed among the participants. Thus, family relationship was negatively affected. Several men slept separately from their spouse because of frequent nocturia. One man even claimed that his spouse blamed him for frequent urination.

We sleep in different rooms. I feel troublesome because I always have to go to the toilet. This is inconvenient for both of us. (P21, severe LUTS)

Not all participants actively responded when asked about the effect of LUTS on their sex lives. Seven participants reported diminished sexual activities even before the LUTS onset, and eight participants reported being sexually active. Regardless of the status of their sex life, all participants acknowledged a decline in sexual desire and functioning as a result of ageing, particularly for elderly men with multiple co-morbid conditions. Three participants believed that LUTS was associated with sexual dysfunction.

I do not have any sexual desires anymore, and it is difficult for me to get an erection during sexual activities. (P13, severe LUTS)

I think that the decline in the ability to urinate and the decline in sexual ability are related. (P14, moderate LUTS)

#### 1.3. Psychological responses towards LUTS

Majority of the participants (*n* = 15) reported that LUTS exerted no psychological effect because they believed that LUTS was a consequence of normal ageing and a process of ‘letting nature take its course’.

No, no, no. I do not think this (LUTS) problem has any impact on me. The only inconvenience is the need to go to the washroom more frequent than usual. I do not think it is a big problem. I do not even mention this to my family (P3, moderate LUTS)

I do not feel guilty. LUTS are natural. Most elderly people have problems as they grow older. Staying perfectly healthy is unrealistic. I do not feel embarrassed when nature takes it course. Going to the toilet frequently is a sign of old age, and it is an inevitable deterioration. (P5, severe LUTS)

Every older friend of mine has health issues, and there is no way for us to stop aging. We are bound to have some health problems. (P8, moderate LUTS)

This problem does not cause me big trouble. The only thing I would say is….I often go to Guangzhou (city of China). I would not take the bus going there anymore. Rather, I like to take the train, which has the toilets in it. (P24, moderate LUTS)

Some participants (*n* = 7) experienced negative emotions associated with LUTS. They reported being embarrassed when they had to urinate frequently during social activities and when they experienced incontinence at various instances.

LUTS have caused a bit, but not a lot, of embarrassment. Sometimes I would be laughed at when I need to go to the bathroom amid a meal with friends. (P4, moderate LUTS)

It makes me feel bad to walk away to use the bathroom when my friends are gathered and having fun. Like in yum cha (Chinese style brunch tea), everybody is interacting happily, and walking away alone makes me feel isolated. (P15, moderate LUTS)

One participant disclosed that he became less confident, isolated himself and felt powerless over his body and surroundings due to his LUTS.

I could no longer travel long distances after having LUTS. This affects my self-esteem. My friends invited me to go out of the country, and they would even pay for my expenses. However, I did not accept their offer because of LUTS…I had many friends when I was young. After I retired, I isolated myself…I feel like I can do little for my body or the environment around me. (P15, moderate LUTS)

A few participants were worried about the progression of symptoms. Their concerns stemmed largely from their fear of developing urinary incontinence and consequently becoming dependent on others for the rest of their lives.

Patients in hospitals may not get out of bed freely. So, they must poo and pee in bed…. It is distressing to have somebody else take care of your excretion. (P11, moderate LUTS)

### 2. Perceptions and misconceptions

#### 2.1 Influenced by social media

The knowledge of the participants about LUTS originated primarily from newspapers and the Internet and partly from attending health talks in elderly centres. Although they were keen on learning how to lead good lives with LUTS, they seldom sought information from health professionals.

When I learned about the effect of lycopene on alleviating LUTS from the newspaper, as well as the Internet, I tried it to see if it would work or not. It was the information that I searched on my own. (P2, moderate LUTS)

I tried lycopene tablets because of the advertisement on the newspapers. (P3, moderate LUTS)

#### 2.2 Misconceptions of LUTS

Majority of the participants lacked knowledge about LUTS, and some even admitted that they had no idea about the topic. Some of their conceptualised causes and nature of LUTS showed a certain degree of misconceptions. For example, some of them believed that LUTS was attributed to natural ageing and essentially untreatable.

I think it is normal. These problems arise as we become older. They are untreatable, and will continue like this. The function has already declined. It cannot be treated. (P22, severe LUTS)

Two participants related LUTS to cancers. One of them believed that pain indicated cancer, and the other believed that LUTS was exclusive to men and equivalent to benign prostatic hyperplasia (BPH) and potential cancer.

It is called prostate, BPH…and may lead to cancer and tumor. (P1, moderate LUTS)

One participant expressed his belief about the causal effect of frequent sexual activities on one’s prostate problems.

Men’s prostate (problems) are related to their sexual activities, but it does not mean that sexual activities are harmful. (P3, moderate LUTS)

Two participants associated LUTS with their dietary habits, such as eating ‘*liang-ye*’, which are dishes that are ‘cool’ in nature based on the teachings of traditional Chinese medicine (TCM).

If I ate some ‘liang-ye’, I would urinate frequently in the day time and at night. Nothing happens when I do not eat it. (P7, moderate LUTS)

One participant considered an unhealthy lifestyle as a possible reason for LUTS.

Nothing is more important than a healthy lifestyle, including keeping fit, not smoking and drinking alcohol, as well as not having an affair outside of marriage. For eating habits, you should take care of it. It (healthy lifestyle) is also associated with a positive mind because depression would worsen the problem. (P4, moderate LUTS)

### 3. Management of LUTS

#### 3.1 Attitudes towards Western treatment approaches

LUTS affected the participants in different ways, but eight participants never sought medical consultation for their LUTS problem because of various reasons. Ten participants mentioned that seeking medical help was unnecessary because the symptoms were tolerable at the moment. Three participants expressed reluctance to see doctors because they were worried that they would be prescribed with additional medications.

I did not go to a urologist because [the impact of LUTS on] my daily life is acceptable. I did not even discuss my symptoms with my general practitioner because they are not very severe. (P17, moderate LUTS)

I do not want to see a doctor for my urinary problem because I am already taking many medications, including those for blood pressure and high blood sugar and cholesterol level. I am already taking so many medicines. I would rather adjust my lifestyle and diet [for LUTS]. (P1, moderate LUTS)

Only one participant received operation for LUTS, and three received Western medication for this problem but quit due to ineffective treatment or worried about side effects of treatment. Two participants strongly opposed using surgical approach for dealing with LUTS for fear of a Foley catheter or that the treatment might recur after surgery. However, a significant number of participants (*n* = 10) expressed that if LUTS deteriorates, they would prefer receiving Western treatment instead of other treatment approaches, such as TCM.

I think the effect of the western treatment is so so…it is better than taking nothing….I always think that these drugs may have some side effects on my other organs…I have this thinking probably due to my innocence to these drugs. (P7, moderate LUTS)

I feel embarrassed if there is a tube (Foley catheter) inserted on me after operation…I am truly frightened if it occurs on me. Also, I know someone whose (LUTS) condition recur even after surgery. (P12, severe LUTS)

If I cannot pass urine one day, I will certainly go to the medical doctor and seek for treatment. I will not consider going to TCM doctor because the treatment effect comes slower. (P17, moderate LUTS)

#### 3.2 Seeking alternative treatment approaches and dietary manipulation

Although most of the participants sought Western medical advice, some of them sought alternative treatments, such as TCM, phytotherapeutic agents and supplements.

It is controlled by (Western) medicine, but the low dosage is already intolerable. I asked the Chinese medicine practitioner for (Chinese) medicine on my own. Otherwise, I cannot void. (P23, severe LUTS)

My neighbor recommended me to use lycopene. It is quite expensive, and I bought it from the chemist. I tried it for several months but find ineffective (P5, severe LUTS)

For some participants who strongly believed in the association between diet and LUTS, they either avoided taking ‘*liang-ye*’ or ate foods that could help alleviate LUTS. Nearly half (*n* = 11) of the participants claimed that they adopted dietary manipulation. Tomatoes and other food products were mentioned.

I think drinking too much herbal teas (one type of ‘liang-ye’) is the reason why I urinate so many times. When I stop drinking these herbal teas, my condition gets better. (P7, moderate LUTS)

I am fond of eating a lot of tomatoes. I eat a lot of tomatoes when there are tomatoes in the dishes. Sometimes, I buy a lot of pumpkin seeds and white melon seeds to eat. They are good for improving LUTS. (P4, moderate LUTS)

#### 3.3 Self-management strategies and lifestyle modification

Instead of actively seeking medical help, some of them chose to adopt self-management strategies to prevent the increasing severity of their LUTS. Most of the participants attempted to cope with the problem by making certain changes in their lifestyle.

Only two participants mentioned exercise to maintain health and cope with the problem.

Doing more exercise makes me feel more energetic. It helps the situation. (P2, moderate LUTS)

Most of the participants reduced urinary frequency through fluid restriction.

I dare not drink too much tea. I also dare not drink water a few hours before sleeping or going out. (P21, severe LUTS)

In addition to lifestyle modifications, the participants used other strategies to manage LUTS. One man believed that staying positive could help in dealing with the situation, and another sought comfort by comparing himself with others.

To be honest, when I go to the toilet, I see people standing to urinate, but some of those who are the same age as me are still standing there long after I have finished and am about to leave. This is why I comfort myself with the knowledge that my condition is not that bad. (P24, moderate LUTS)

Finally, some comments demonstrated cognitive manipulation to adapt to the problem. Two participants did not think about the problem, and one felt good by not thinking about it or by just ‘letting nature take its course’. Two others mentioned that the use of willpower could control urinary frequency.

I have not prepared any special diet for my health. I did not even try beneficial foods recommended by others. Just let nature take its course. (P5, severe LUTS)

Sometimes, it is about willpower. If you are psychologically prepared, the time between urination can be longer. (P1, moderate LUTS)

Majority of the participants attributed LUTS as a natural ageing problem and did nothing about them.

## Discussion

### Effect of LUTS

Urination difficulties were manifested among the participants in several ways. Some men had to void often and went to the toilet as soon as they felt the need to void. Otherwise, they would wet their trousers. Some men also reported a sting in the urinary tract and dribbling. Almost half of the participants suffered from nocturia, which affected their sleeping quality.

According to the participants, LUTS affected their social activities and led to their avoidance of social situations, long-distance travel and other events that lacked easily accessible toilets. This observation matched the findings of Pinnock et al. [18], who conducted 19 focus group studies in South Australia. They found that access to toilets in a shopping centre or a bus stop can determine whether men with LUTS would participate in outings. In a hermeneutic phenomenological study in the United Kingdom, the participants expressed embarrassment for having to go to the toilet regularly, particularly when socialising in the presence of family and friends [13].

Most of the participants were unwilling to seek social support and even embarrassed to share this topic with their peers. This phenomenon was also observed in people from other countries. Men in South Australia were reluctant to talk about their health, particularly those topics that pertain to urinary or sexual problems [18]. In a Korean study, approximately 42.7% of community-dwelling elderly with BPH never consulted anyone about their symptoms [21].

Contrary to Western literature, majority of the participants in our study reported that LUTS showed minimal psychological effect, and they accepted these symptoms. They considered LUTS as part of the normal ageing process and a process of ‘letting nature take its course’. This finding may be governed by the underlying traditional Chinese philosophy that sickness is part of life [30]. The cultural stereotype of the elderly as individuals who suffer from multiple diseases may further affect participants’ perception that LUTS is normal among ageing males. These findings are consistent with those of Chinese cultural studies, which indicated that individuals accept their diseases and embrace their fate [31]. Chinese naturalistic philosophy based on Taoist principles may influence the perceptions and actions of elderly Chinese men [32]. Birth, ageing, sickness and death are viewed as parts of one’s lifecycle that nobody can escape. People should live in harmony with nature and accept the natural process of life [31].

Even though many participants claimed that LUTS was part of normal ageing and accepted it as a nature of life, various emotional distresses, which included self-blaming and the feelings of being troublesome, miserable, hopeless, sad and embarrassed, were also reported. In the current study, LUTS severity was significantly correlated with life quality as reflected in the generally unsatisfactory QoL of the interviewees (mean = 3.17). Urological symptoms may exert adverse effects on the psychological well-being of men by affecting their emotions. These conditions reduced the functions and QoL of patients [8,12,21,33]. Schulman et al. [34] suggested that sleep disruption negatively affects mental health, reduces work performance, causes memory impairment and induces depression. In a large prospective cohort of 2000 Chinese men in Hong Kong, moderate-to-severe LUTS was significantly associated with increased odds of having clinically relevant depressive symptoms even after adjustment [35].

Although the pathogenesis of the relationship between LUTS and erectile dysfunction is unclear, various studies reported that sexual disorders increase with age and LUTS severity. When the participants in the current study were asked about the effect of LUTS on their sexual lives, only three participants mentioned the relationship between LUTS and sexual function. This result suggests that sexuality is a sensitive and embarrassing topic among the elderly Chinese population and is difficult to discuss openly. Despite the prevailing recognition in Western society that LUTS adversely affects the sexual lives of the male population [36–38], half of the participants in our study already stopped having sexual activities even before the LUTS onset. The remaining number of participants reported being sexually active, with very few reporting sexual dysfunction. In Chinese culture, men accept decreased sexual desire or sexual dysfunction as part of ageing [39]. An active sexual life is considered inappropriate, particularly for the elderly. Traditional Chinese philosophies view sexuality as only a function of procreation rather than a source of pleasure or enjoyment [40]. This view may explain the discrepancies between our findings and those of Western populations. In addition, several participants reported sleeping separately from their spouses to minimise the disturbance caused by nocturia. This finding may be a distinct feature of Chinese couples or a coping method to maintain spousal relationship when problems arise.

### Perceptions and misconceptions

The current study indicated that most older Chinese men in Hong Kong lacked the knowledge or even held misconceptions about LUTS. Some of them believed that LUTS was caused by natural ageing and untreatable. A few related LUTS to cancers and even believed that frequent sexual activities caused prostate problem.

Misconceptions among the elderly men are also common in other countries, such as Korea [21] and Spain [41], wherein a high proportion of affected men do not seek medical advice because they believe that LUTS is part of natural ageing. Thus, seeking medical help is unnecessary. This result reflects the low rates of medical help consultation found in other studies because of this misconception [20,40]. In the present work, one participant believed that LUTS was the same as BPH, and another participant believed that BPH was directly related to cancer. In a qualitative study conducted in the United States, 67% of the participants believed that BPH (a common cause of LUTS) and cancer are associated; BPH surgery is performed for cancer removal or BPH leads to cancer [42]. One participant believed that LUTS only occurs in males. The present study explicitly indicated that many older Chinese men may possess limited knowledge about LUTS. Therefore, health educational interventions should be provided to instill correct knowledge, empower the development of customised coping strategies and encourage patients to seek medical consultation and care from health professionals for their LUTS. In a randomised control study among 109 BPH patients, patient counselling was able to significantly improve the knowledge, symptoms, management, treatment outcomes and QoL of patients [43].

### Managing LUTS

A number of participants (*n* = 10) expressed that if LUTS deteriorates or when some intolerant symptoms arise, they would prefer receiving Western treatment instead of other treatment approaches. However, their failure to detect symptoms that may indicate a serious condition and their tendency to delay seeking medical attention can lead to kidney disease, urinary tract infections and bladder damage [14]. In a qualitative study conducted in Finland, pre-operative medical investigations, endoscopy and decision making with regard to treatment are anxiety provoking and difficult [44]. Therefore, health-care professionals should provide these patients with sufficient knowledge and offer consultations to encourage them to participate in the decision-making process.

In this study, majority of the participants sought alternative treatments, such as TCM, special voiding positions and phytotherapeutic agents and supplements, including lycopene tablets and dietary consumption of tomatoes, pumpkin seeds and white melon seeds. Given that lycopene belongs to the carotenoid family and mostly exists in nature [45], we found that participants in this study preferred to consume great amounts of tomatoes in their diet. However, scientific evidence about the role of lycopene in combination with *Serenoa repens* (a phytotherapeutic agent) in the management of LUTS secondary to BPH remains limited [46]. Therefore, further studies should be conducted to gather scientific evidence that supports the effectiveness of these supplements in managing LUTS.

A number of participants believed that the consumption of ‘*liang-ye*’ may cause frequent urination. Since the ancient times, TCM practitioners have recognised dietotherapy and used the ‘warm’ and ‘cool’ nature of food to achieve body equilibrium [47]. From the TCM perspective, frequent urination and dripping urination may be due to ‘deficiency cold’ of the *lower jiao* (pelvic region) [48]. Therefore, further consumption of ‘*liang-ye*’, such as green tea, melons, soy beans and pears, may aggravate LUTS. Meanwhile, difficulty in urination, dripping urination and pain in micturition may be caused by ‘stagnation of damp heat’ in which food in ‘cold’ nature may be beneficial [48]. Therefore, rehabilitation with diet should be guided by the differentiation of syndromes and correct diagnoses if TCM principles are to be followed.

One participant shared his successful experience of using a specific voiding posture (i.e. standing with heel raised during voiding) for complete bladder emptying. Previous studies did not specifically investigate this specific voiding posture. However, a recent systematic review indicates the influence of voiding position on the urodynamic profile of patients and reported that sitting position can significantly decrease the post-void residual urine volume in patients with LUTS [49]. Standing with heels raised may require abdominal muscle contraction to maintain balance. This posture can increase intra-abdominal pressure and influence post-void residual urine volume. Therefore, further research regarding the effects of different voiding positions and their effects on the urodynamic profile of patients should be conducted.

Most of the participants tried to reduce urinary frequency through fluid restriction. Elstad et al. [50] revealed that fluid manipulation is a common self-coping strategy used by individuals with LUTS. Nevertheless, improper fluid restriction may negatively affect the health of patients. Therefore, individuals with LUTS may need guidance in fluid management. In addition, several participants mentioned performing exercises as a method to improve their LUTS status. This method is a possible coping strategy for people who have LUTS. A recent cross-sectional study conducted by Fowke et al. [51] showed that high energy expenditure during leisurely physical activities and light housework activities is significantly associated with low LUTS severity. Surprisingly, some men cope with LUTS using their willpower to control urinary frequency, cognitively ignore the problem or maintain a positive mood to alleviate their problems.

### Limitations and future directions

The major limitation of this study was that the subjects were recruited only from the community centres in which majority of the participants were not receiving treatment using medical or surgical approaches. Thus, the profile of participants was not varied enough to obtain data saturation on the overall experience of ageing males with LUTS. As the participants were all elderly men, the exploration of sensitive areas, such as the effect of LUTS on sexuality in some of the participants, was hindered because of their hesitation to share with young female interviewers. Future studies may consider extending the subject recruitment in other settings (e.g. hospitals and urological clinics) to understand the motivation and challenges of those patients who have adhered to active treatment to manage this problem.

### Conclusion

The life experiences of older Chinese men with LUTS indicated that this condition could adversely affect the physical aspects of their daily lives. Most of the participants were unwilling to seek social support and even embarrassed to share this topic with their peers. Nevertheless, majority of the participants reported that LUTS exerted no psychological effect because they believed LUTS was a consequence of normal ageing and in ‘letting nature take its course’.

Most of the interviewees lacked knowledge about LUTS and held misconceptions about the condition. This limitation prevented them from pursuing medical aid. Most of the participants also sought alternative treatment and developed self-help methods to cope with the symptoms. The strategies employed by the participants included use of Chinese medicine, supplements, dietotherapy, specific voiding posture, fluid restrictions, exercise and the use of willpower to alleviate the problem. The findings of this qualitative study can raise awareness about the life experiences, perceptions, misconceptions and health-seeking behaviour of ageing Chinese males with LUTS. This study can provide proper health education and advice for this population group.

## Supporting information

S1 Table Categories of the experiences of community dwelling aging males living with lower urinary tract symptoms. (DOCX)

S2 Table COREQ checklist. (DOCX)
