# Supplementary material for: Qualitative insights into the experiences of living with moderate-to-severe lower urinary tract symptoms among community-dwelling ageing males
Source: PLoS One. 2017 Oct 30;12(10):e0187085. doi: 10.1371/journal.pone.0187085 (PMC5662182; doi:10.1371/journal.pone.0187085)
Supplement: S1 Table — (DOCX) [file pone.0187085.s001.docx]

S1 Table

Categories of the experiences of community-dwelling aging males living with lower urinary tract symptoms

| **Categories** | **Main themes** |
| --- | --- |
| 1. Impact of LUTS | - 1. Frequent uncontrolled urinary habit   2. Avoidance of social activities and a strain on relationship   3. Range of psychological responses: Taking it as a natural course to losing self-esteem |
| 2. Perception and misconceptions | - 1. Influenced by social media   2. Misconceptions toward the problem |
| 3. Managing LUTS | - 1. Attitudes toward Western treatment approaches   2. Seeking alternative treatment approaches and dietary manipulation   3. Self-management strategies and lifestyle modification |
